# Curses in Acts: Hearing the Apostles’ Words of Judgment Alongside ‘Magical’ Spell Texts

**DOI:** 10.1177/0142064X17703296

**Published:** 2017-04-20

**Authors:** Benedict H. M. Kent

**Affiliations:** Department of Religions and Theology, University of Manchester, UK

**Keywords:** Acts, apostles, curse, Coptic, magic, papyri

## Abstract

Scholars of Luke–Acts have struggled to define the apostles’ proclamations of judgment on those who threatened the early Christian community. Ananias and Sapphira (Acts 4.32–5.11), Simon magus (8.4-25) and Bar-Jesus (13.4-12) all fall victim to the apostles’ words of power, yet scholars have typically shied away from categorizing their speeches as curses. Close analysis of the structure, style, phonaesthetic and dramatic aspects of the Greek texts suggests, however, that Luke indeed intends the apostles’ speeches to be heard as curses whilst simultaneously presenting them as legitimate acts of power. A comparison with Greek and Coptic ‘magical’ texts helps to place the curses of Acts in the context of cursing traditions in the wider ancient Mediterranean world.

## Introduction^1^

Scholars of Luke–Acts have reached little consensus over how to define the apostles’ proclamations of judgment against those who threatened the early Christian community. They have generally regarded Paul’s words to Bar-Jesus as a form of a curse,^2^ but few scholars have identified the speeches against Simon magus and Ananias and Sapphira in the same terms, despite their similarities with the Bar-Jesus episode.^3^ Some explicitly deny the curse-like invectives in Peter’s speech to Simon magus.^4^ Others portray his speeches to Ananias and Sapphira as prophecies rather than curses,^5^ or else relocate responsibility for the two deaths elsewhere.^6^

This article, in contrast, will employ literary analysis to demonstrate key links between these three episodes in Acts, arguing that these conflict narratives should be read in continuity with each other.^7^ An analysis of each episode will, in the first place, attend to the thematic, grammatical and phonaesthetic properties of the apostle’s speech to highlight its dramatic power in a performance setting, both within the narrative and in the narrative’s recitation amongst Christian communities. Secondly, in each case I will employ a comparative perspective to evaluate the curses of Acts in relation to a selection of Greek and Coptic ‘magical’ texts. This approach aims to demonstrate how an ancient audience of Luke–Acts would have heard the apostles engaging with curse traditions typical of ancient Mediterranean culture while also viewing them as divinely authorized acts of power superior to acts of ‘magic’.

The hesitancy of scholars to categorize the apostles’ judgments as curses may be linked to the continued association of the label ‘curse’ with uncritical conceptions of ‘magic’ that define magic as some impure or primitive form of religion.^8^ In fact, curses play an important role in the Jewish and Christian scriptures.^9^ In this article I will follow the more anthropologically informed definition of a curse employed by David Frankfurter in his analysis of blessings and curses in Roman Imperial and Late Antique Egypt (2005: 158). Frankfurter describes a curse as ‘a ritual performance that transfers subversive power to some object, *or* the subversive power that plagues one following such a performance (as in “her curse still rests on me”)’. However, I would also add the qualification that this subversive power can be operated in a way deemed to be socially legitimate or illegitimate. Thus I use ‘curse’ as a neutral term that is open to construal as either ‘miracle’ or ‘magic’ according to its performer’s perceived authority and status. Following the studies of Poupon, Reimer, Marguerat and others,^10^ this article will be sensitive to the strategies employed by the author of Acts to navigate a context in which the apostles’ words of power are susceptible to accusations of magic. It will go further than previous studies by placing the strategies themselves within cursing traditions of Late Antiquity, showing greater overlap between them than previously acknowledged and the potential tensions in the claim to distinction found in Acts. Given that scholars widely accept Paul’s words to Bar-Jesus to be a form of a curse, we will begin with that episode, identifying the key features of the curse itself before progressing to discuss the earlier episodes in the narrative.

## Bar-Jesus (Acts 13.4-12)

The beginning of Acts 13.4-12 sets the scene in Gentile Salamis and introduces some of the key themes that are to follow in the narrative. It employs intertextual allusions in order to parallel Paul with Bar-Jesus and to posit the pair as natural opponents. Paul is ‘sent out by the Holy Spirit’ (13.4),^11^ in a similar manner to the OT prophets (1 Kgs 18.46, for example). Bar-Jesus is described as a ψευδοπροϕήτης (13.6), the prophets’ natural antagonist.^12^ Paul is ‘*filled* with the holy spirit’ (13.9) whereas Bar-Jesus is said to be ‘*full of* all deceit and villainy’ (13.10). Such rivalry is reminiscent of the prophetic clashes attested throughout the Jewish scriptures, none more famous than Elijah’s challenge to the prophets of Baal (1 Kgs 18.1-40). The text also describes Bar-Jesus as μάγος, which is reminiscent of Moses and Aaron’s encounter with the magicians of Pharaoh (Exod. 7–9). These intertextual frames prepare the audience to expect a power conflict between Paul and Bar-Jesus.

Paul’s speech can be divided into the following parts and contains three themes that are also found in Peter’s pronouncements in Acts 4.32–5.11 and 8.9-25.

**Table:** 

13.10a	ὦ πλήρης παντὸς δόλου καὶ πάσης ῥᾳδιουργίας, υἱὲ διαβόλου, ἐχθρὲ πάσης δικαιοσύνης,
	(Insight and re-labelling)
13.10b	οὐ παύσῃ διαστρέϕων τὰς ὁδοὺς [τοῦ] κυρίου τὰς εὐθείας;
	(Accusation in the form of a rhetorical question)
13.11a	καὶ νῦν ἰδοὺ χεὶρ κυρίου ἐπὶ σὲ καὶ ἔσῃ τυϕλὸς μὴ βλέπων τὸν ἥλιον ἄχρι καιροῦ.
	(Verdict)

Paul begins by re-labelling his opponent. He then asks a rhetorical question which provides an effective contrast to his previous statements. His speech comes to a climax as he vividly curses his opponent by ‘the hand of the Lord’.

Paul’s pronouncement is notable for its direct style, created by its repeated re-labelling of the adversary. In addressing Bar-Jesus (‘son of Jesus’) as υἱὲ διαβόλου (13.10), Paul employs irony to accuse him of being an impostor (Barrett 1994: 617). In calling him ἐχθρὲ πάσης δικαιοσύνης, Paul confirms Luke’s description of Bar-Jesus as a false prophet. In re-labelling his opponent, Paul both reveals his opponent’s true identity and asserts his dominance over him.

The first of these three themes is the presence of the demonic, introduced through the re-branding of Bar-Jesus as υἱὲ διαβόλου. This designation is a major element in creating Paul’s direct style of address. Its position in the syntax of the sentence also emphasizes deceit and villainy and possibly implies that the label ‘son of the devil’ is dependent on or deducible from Bar-Jesus’ actions.

The second theme, implicit in this instance, is the adversary’s loss of status in the community of God. Despite appearing to be a ᾽Ιουδαῖος, the text reveals that Bar-Jesus is in fact a ψευδοπροϕήτης (13.6), effectively undermining his legitimacy as a member of the house of Israel. In fact, by describing him as a μάγος Luke depicts Bar-Jesus as someone more akin to an antagonistic Gentile. In addition to identifying him as a μάγος, the opening verses (6-7) characterize him in a fashion strongly reminiscent of the court magicians in the Jewish scriptures who attend the palaces of Gentile kings (ὁ σοϕός in Gen. 41.8; ὁ μάγος in Dan. 2.2). Such intertextual allusions contribute to the re-labelling of Bar-Jesus as an outsider in relation to the community, as impious and atheistic as a Gentile magician. Paul’s curse completes the stripping of Bar-Jesus’ status as a Jewish holy man when he challenges him (13.10): οὐ παύσῃ διαστρέϕων τὰς ὁδοὺς [τοῦ] κυρίου τὰς εὐθείας; Paul effectively accuses his opponent of perverting the nation, an accusation traditionally made against those who promoted idolatry and impurity in the OT (Exod. 32.7; Deut. 32.5; Num. 15.39; Ezek. 14.5) and for which dramatic punishments were commanded (Malina and Neyrey 1991: 110). Paul publicly denounces Bar-Jesus’ status within the community of God and his curse functions as an excommunication.

The third theme is the impurity of the adversary’s heart. Paul describes Bar-Jesus as ‘*full of* all deceit and villainy’ (13.10). Paul’s searching gaze (13.9) reveals that Paul has insight into his adversary’s inner self, an ability commonly associated with God and his prophets in the OT, as well as with Jesus in the gospels.^13^

Finally, the reaction of the witness to Paul’s miracle is also significant: ‘When the proconsul saw what had happened, he believed’ (13.12). Paul’s curse, like his more positive acts of power, leads to conversion or else a newly found respect for the Christian movement.

### Comparative analysis

Historical sources such as papyri, *defixiones*, amulets, ostraca and inscriptions provide valuable insights into the practice of cursing in ancient Mediterranean societies.^14^ Whilst the dating of the Coptic Christian curses in Meyer and Smith’s collection varies widely (first century ce to eleventh or twelfth century), the texts in Preisendanz’s *Papyri Graecae Magicae* (*PGM*; 1973–74), from the second century bce to fifth century ce, bring us closer to the New Testament period. Binding spells in particular appear to have been widely known in the ancient world (Gager 1992: 243-64), and in recent years they have received significant academic attention after periods of neglect.^15^ Despite these texts being dated to a range of historical periods,^16^ Ritner suggests that Egyptian ‘magic’ in the Roman era largely followed traditional methods and function. Furthermore, the same ‘mechanics’ operate in Demotic and Greek magical texts.^17^

Older treatments of binding curses/*defixiones/*κατάδεσμοι might suggest a practice hardly comparable to the words of the apostles in Acts.^18^ For one, they reflect a different cultural setting from that of Luke–Acts, showing a greater degree of religious syncretism in their appeals to pagan, Jewish and Christian deities. Older binding spells are highly formulaic, using cognates of the verb καταδέω, and later spells are filled with ‘mystical’ vocabulary (Gager 1992: 5-7). They also suggest a different performance setting: they were performed in private, addressed to a deity or a spirit and often deposited in tombs or buried under houses (Gager 1992: 18-21). The circumspect nature of their performance, their attitude towards divine power, and their appearance in certain handbooks often lead to binding spells being labelled amongst ‘magical’ practices.^19^ In contrast, the apostles perform their curses in public, towards the accused, and are often described with the aid of the religious categories of ‘prophetic utterance’ or ‘punitive miracle’.^20^

However, the recent publication of Coptic and Greek spells demonstrates that their performers rarely use their native terms for ‘magic’ or ‘sorcery’ and do not consider their actions to be operating within such spheres (Meyer and Smith 1999: 2). Scholars of binding curses have also added more variation and detail to their classifications of the texts in order to reflect the diversity of the emerging evidence. Binding spells are now judged to fall into three categories.^21^

1. Direct curses, addressed to the intended victim, often using a simple formula such as ‘I bind X’.
2. Prayer wishes, addressed to a deity, supplicating or commanding it to bind the victim using second- or third-person imperatives such as ‘Bind X!’^22^
3. Persuasive analogies, often accompanied by figurines to ‘persuade’ the intended victim to adopt the attributes of a dissimilar object. For example, ‘As this lead is cold, may X become cold.’

Faraone places these curses in various agonistic contexts, such as sporting or performance events, legal cases and amorous or commercial relationships (1991: 10-17). Whereas binding curses from classical Athens are very formulaic, curses of the imperial era are far more elaborate and explicit. In addition to *voces magicae*, curses typically employ ‘repetition, pleonasm, metaphor and simile, personification, rhythmic phrases, exaggeration, threats, promises, prayers and formal appeals’ (Gager 1992: 13-14). I would also add to this list quotations from sacred scripture and elements of historiola. Earlier binding spells often relied on recognizable formulae, but later binding spells and other forms of curses could be expressed in varying forms.

Versnel has made a compelling case for a seemingly related but separate category of curses, which he terms ‘prayers for justice’ (1991a). Such curses are distinct from traditional *defixiones* in the way that they often address the local gods of the institutional cults, in their lack of magical formulae and their use of quasi-legal language, in their supplicatory posture, and in their desire for recompense on named enemies rather than anonymous ones (1991a: 90). Despite appearing to have been particularly popular in the third and fourth centuries ce, especially in the Latin West of the Empire,^23^ these ‘prayers’ also appear in the Greek East from the Hellenistic period onwards (1991a: 68-81). However, no category of curse is absolute, and we observe elements of these prayers in other types of curses, which shows how difficult it is to separate ‘magical’ binding spells from ones which might appear more ‘religious’.

This is not to downplay the differences between these collections of curses and the apostles’ judgments in Acts.^24^ The private social context attributed to the majority of the binding curses, combined with their use of ritual action (twisting, contorting, impaling and burying the lead tablet and/or figurine), constitute a type of curse distinct from that of public cursing (Gager 1992: 26). For some, the private or antisocial context and ritual action that accompanies the verbal curse is *the* reason why the practice should be designated a ‘magical’ act (Audollent 1904: xl-xliii; Graf 1997: 128). In contrast, the public setting of the apostle’s curses presents them to their readers as socially legitimate, even functioning as deviancy labels that alter the public’s perception of the accused (Malina and Neyrey 1991: 99-100).^25^ However, whilst *defixiones* constitute the private practices of ancient individuals, they employ many of the same techniques as public forms of cursing and are thus useful for purposes of comparison (Gager 1992: 26). Certain tablets suggest public display^26^ and share a number of features with curses performed on behalf of Greek cities and communities attested in inscriptions (Faraone 1991: 9). Versnel even suggests that some ‘prayers for justice’ might have also been performed publicly so that the accused might have known of their condemned status before the divine (1991a: 81, 90).

Some recent commentators on Acts have remarked on the similarities between the apostles’ judgments and ancient cursing practices.^27^ The following analysis highlights not only their common motifs and stylistic features, but also their common agonistic context.

At first glance, Paul’s judgment on Bar-Jesus appears the most curse-like because of his direct style of address and use of particular motifs. His use of the second person imperative (13.11a: καὶ νῦν ἰδοὺ) draws the comparison to the mode of Faraone’s first category of ‘direct curse’, ‘which is a performative utterance, that is, a form of incantation by which the *defigens* hopes to manipulate his victim in an automatic way’ (Faraone 1991: 10). Although Paul does not say ‘I bind you’, nor utter any of the customary expressions of urgency in the Greek curse texts (such as ἄρτι, ἄρτι, ἤδη, ἤδη), he does declare emphatically ‘and *now* listen … you will be blind’, which is followed by an automatic effect, παραχρῆμά and the participle περιάγων (13.11), suggesting the immediate fulfilment of Paul’s pronouncement.^28^

Paul’s utterance also shares a number of stylistic features and motifs with the curse texts. Paul’s use of epiplexis in his curse on Bar-Jesus (οὐ παύσῃ διαστρέϕων τὰς ὁδοὺς [τοῦ] κυρίου τὰς εὐθείας;) mirrors the style of some Greek curse texts that employ epiplexis seemingly as part of their rhetorical strategy. *PGM* IV.467-68 and IV.831-32 quote Homer’s *Iliad* 8.424 in the form of a question.

τολμήσεις Διὸς ἄντα πελώριον ἔγχος ἀεῖραι;

Will you dare to raise your mighty spear against Zeus?^29^

Devices such as rhetorical questions serve to heighten the drama of the curse ritual and, if performed publicly, had the potential to intimidate one’s opponent.

The most obvious parallel between Paul’s curse and Greek and Coptic curse spells is the desire for the deity to strike the spell-caster’s adversary. *PGM* XIVc.16-27 uses καταβάλλω to convey sudden, harmful action as well as a spatial dynamic of descent.

ἧκέ μοι κ[αὶ] βάδισον καὶ κατάβαλε τὸν δεῖνα (ἢ τὴν δεῖνα) ῥίγει καὶ πυ|ρετῷ αὐτὸς ἠδ[ί]κησέν με καὶ τὸ ἇιμα τοῦ Τυϕῶνος ἐξέχυσεν παρ᾽ ἑαυ|τῷ (ἢ αὑτῇ). διὰ το[ῦ]το ταῦτα ποιῶ

‘Come to me and go and strike down him, NN (or her, NN) with chills and fever. That very person has wronged me and he (or she) has spilled the blood of Typhon in his own (or her own) house. For this reason I am doing this’ (*PGM* XIVc.25-27 = *PDM* xiv.690-94, trans. Hock and Johnson [1992]).

Such requests are common in Coptic spell texts. One speaker orders the deity to ‘strike Philadelphe and her children’ (*ACM* 29, trans. Meyer). Another implores: ‘You must strike Prestasia and Tnounte and Eboneh, quickly, deservedly’.^30^

Many curse texts also emphasize the spatial movement of descent. They speak of bringing their victims ‘down’ in some manner (Graf 1997: 121-23),^31^ are themselves often buried in tombs and wells, and implore spirits of the dead or chthonic deities. For example, *ACM* 88 commands: ‘You must bring [them] down from their heights …’.^32^ Acts likewise highlights the spatial movement of curses quite literally through its depiction of Bar-Jesus as struck by the hand of God. In fact, Acts could be said to foreground the spatial dynamic of Paul’s curse and its effect. The repeated use of ἐπί in the words καὶ νῦν ἰδοὺ χεὶρ κυρίου ἐπὶ σὲ̀ and παραχρῆμά τε ἔπεσεν ἐπʼ αὐτὸν ἀχλὺς καὶ σκότος depicts the hand of God as above and falling upon Bar-Jesus, followed by the darkness by which he is smitten (13.11). The vivid image of descent conjured for the audience reflects the descent of Bar-Jesus’s social status as he submits to the apostle.

Another parallel between the two groups of texts is their choice of affliction. Multiple Greek and Coptic curse texts seek to inflict blindness upon their enemies. *PGM* III.494-611 appeals to a deity who ‘lowers darkness’.

δεῦρό μοι,|| κύριε, ὁ ποτὲ τ[ὸ] ϕῶς ἀνά[γ]ων, ποτὲ τὸ σκότος κατά|γων <κατὰ> τὴν σεαυτοῦ δῦναμιν […]

‘Come to me, / lord, you who sometimes raise the light, sometimes lower the darkness [with] your own power’ (ll. 564-66, trans. Grese).

Again, this spell evokes a descending spatial dynamic through its use of κατά in κατάγων. Similarly, the prayer in PGM VII.764-65 describes the deity’s ability to ‘wane from light into darkness’ (trans. Grese).^33^ One Coptic curse asks the deity to ‘cause their eyes to fog and come out’ (*ACM* 92, trans. Smith). Another requests blindness to fall on its victim’s ‘two eyes’ (*ACM* 91, trans. Smith).

Both Paul’s curse and the ‘magical’ curse texts also share a sense of victimization: many Greek and Coptic curse texts are characterized by their speaker’s desire for justice after being wronged. *ACM* 28 begins: ‘avenge me on the one who opposes me and on the one who has driven me from my place’ (trans. Meyer). Others present their speakers as vulnerable persons or victims of violence, gossip or slander (*ACM* 89-94). Paul’s curse depicts Bar-Jesus as an aggressor; he is an ἐχθρός, an adversarial term in curse texts.^34^ He is also ‘full of deceit (δόλος)’, a common term for unknown causes of death which Versnel includes as part of an ‘idiom of suspicion’ that characterizes ‘prayers for justice’ (1999: 131-34, 134). However, Luke suggests that it is God who is the victim of Bar-Jesus’ aggression (13.10), which helps to portray Paul’s curse as an act of divine judgment rather than one of personal vengeance.

The curse texts and Acts also propound the belief that the spell-caster’s opponent is possessed by a demon (*ACM* 88, 92). Although the devil is not exactly the same as a demon in the NT, Paul shares the concept that his opponent is filled by a malevolent force. In Acts it seems especially important for the apostles to identify the satan as the force of evil in direct opposition to God (Garrett 1989). However, this concept also has to be balanced with the seemingly jarring evidence that the devil is an instrument of punishment.^35^ Although it is the ‘hand of God’ that strikes Bar-Jesus, it is into darkness that he is cast. Luke has previously used σκότος to indicate a personified malevolent force (Lk. 1.79), which appears to concur with the thought of the Bar-Jesus narrative.^36^ The curse texts also identify the demonic as both present in their enemies and as instruments of punishment. One curse includes the command: ‘Send to him an evil demon’, and another: ‘You must make a demon descend upon her’ (*ACM* 91, 93, trans. Smith). Another text requests that the deity brings physical pain and ‘demonic madness’ on its victim (*ACM* 106, trans. Emmel).

The limited duration of Bar-Jesus’ blindness (13.11) has interested scholars and is often understood to be in keeping with the theological programme in Acts that desires sinners to repent and submit themselves to God’s mercy. Garrett takes an opposing view in interpreting Bar-Jesus’ blindness as the final and *lasting* image of the scene simply because the curse is not relieved within the narrative of Acts (1989: 84). But whilst it is the final image, the words ἄχρι καιροῦ (13.11) prevent it from being the lasting one. At first glance, Paul’s seeming allowance for repentance contrasts with some of the Greek and Coptic curses that command enduring afflictions on their opponents (*ACM* 91, 93, 95, 96). However, Faraone has highlighted how many of the Greek binding curses seek not to harm aggressively but to restrict defensively (1991: 8). *ACM* 106 includes an intriguing clause in its command: ‘Let them come and bring them down upon the body of N. child of N. … until I myself, N. child of N., have mercy on it’ (trans. Emmel). If Faraone’s argument is correct, namely, that Greek binding curses should be primarily characterized by their desire to control their opponents rather than by their will to harm, then Paul’s curse on Bar-Jesus fits on the more defensive end of a spectrum of curse traditions that span from temporarily incapacitating a person, to causing illness and even killing. Luke’s theological perspective does not place the apostle’s speech outside the field of cursing but rather complements the type of curse that is cast.

A final parallel between Acts and the curse texts can be found in their common appeal to well-known miracle stories. A number of the spell texts from late Antiquity employ historiola as a means of accessing divine powers described in stories of past events. The curse texts often appeal to OT stories of God’s judgment against figures such as Cain, Pharaoh or the Assyrian army (*ACM* 90, 90, 95). Luke–Acts also connects to OT miracle stories through allusions. Paul describes the χεὶρ κυρίου working against Bar-Jesus, a probable allusion to Moses bringing the plagues against the Egyptians (δάκτυλος θεοῦ in LXX Exod. 8.15), as well as to Jesus whose exorcisms are described as ἐν δακτύλῳ θεοῦ (Lk. 11.20). Paul’s curse does not employ historiola, but it does allude to OT intertexts that, as well as framing the scene within certain narrative expectations, function to suggest that both Paul and the text of Luke–Acts carry a similar authority to that of the OT. Both Paul’s curse and the spell texts appeal to holy scriptures to acquire some form of status or power. In summary, a comparison of Paul’s curse with Greek and Coptic curse texts has shown that they share a number of characteristics such as demonic possession and punishment, the desire for immediacy, the use of spatial imagery, and connections to historical demonstrations of divine power.

Although there is a shared sense of victimization in the two groups of texts, the curse of Acts 13.4-12 differs in its overall outcome. Whilst the desired outcome of many curse spells is revenge and satisfaction for the spell-caster,^37^ Paul’s curse serves to win the respect and even the conversion of Sergius Paulus.^38^ But for Luke, this reaction shows that the apostles’ curses are not primarily intended to punish but to demonstrate God’s power and the authority of their message. Luke undoubtedly portrays Paul’s words to Bar-Jesus as a curse, but he ensures that it fits within a narrative framework in keeping with his overall theological programme. Intriguingly, this theological programme at times ventures into a specifically ‘religious’ agonistic context where the apostle competes with a Jewish magician for the allegiance of an elite Roman figure.

## Simon magus (Acts 8.4-25)

Peter’s encounter with Simon magus is similar to Acts 13.4-12 in both its thematic and stylistic features. Like the Bar-Jesus episode, Acts 8.4-25 is set outside the familiarity of Jerusalem. Like that episode, the text draws parallels between the apostles and their adversaries to establish an agonistic context. It repeats the word προσεῖχον to show that the crowds ‘listened eagerly’ to Philip (8.6), in the same way that they ‘listened eagerly’ to Simon (8.11), suggesting that the apostle competes for their attention as Paul did with Sergius Paulus. But it also firmly distinguishes between them by clarifying their contrasting sources of power. Philip demands attention through spirit-empowered signs, whilst Simon amazes the people by his ‘magic’. As with Bar-Jesus, Simon’s association with μαγεύω may have reminded audiences of notorious adversaries in the Jewish scriptures.^39^ The text’s techniques of mirroring and contrasting the two figures invite the audience to expect a confrontation between these natural competitors. Furthermore, they reveal the substantial similarities between the magic that Simon practises and the signs that Philip performs.

In this episode, the bite of the curse comes at the beginning of the speech in the form of a sharp rebuke, followed by a series of verdicts and accusations.

**Table:** 

8.20	τὸ ἀργύριόν σου σὺν σοὶ εἴη εἰς ἀπώλειαν
	(Rebuke)
	ὅτι τὴν δωρεὰν τοῦ θεοῦ ἐνόμισας διὰ χρημάτων κτᾶσθαι.
	(Accusation against behaviour)
8.21	οὐκ ἔστιν σοι μερὶς οὐδὲ κλῆρος ἐν τῷ λόγῳ τούτῳ,
	ἡ γὰρ καρδία σου οὐκ ἔστιν εὐθεῖα ἔναντι τοῦ θεου.
	(Verdict and accusation against motivation)
8.22	μετανόησον οὖν ἀπὸ τῆς κακίας σου ταύτης καὶ δεήθητι τοῦ κυρίου, εἰ ἄρα ἀϕεθήσεταί σοι ἡ ἐπίνοια τῆς καρδίας σου,
8.23	εἰς γὰρ χολὴν πικρίας καὶ σύνδεσμον ἀδικίας ὁρῶ σε ὄντα.
	(Instruction to repent and accusation of demonic influence)

Like Acts 13.10-11, Peter’s curse maintains the drama through its direct style of address. The second person pronoun is again heavily repeated, making use of σου, σοί and σε eight times in 8.20-23. This focuses the audience’s attention firmly on Peter’s opponent and indicates that Peter has likewise fixed his gaze on him. In contrast, the double use of the first person pronoun in Simon’s response to Peter shows Simon submitting to the direction of Peter’s attention and verdict.

The phonaesthetic features of Peter’s curse also function to heighten the drama of its performance. The text employs sibilance in σου σὺν σοὶ and εἰς. It also creates assonant effects with repeated ει and η sounds (εἴη εἰς ἀπώλειαν). Such devices give a fluid, poetic quality to phrases like τὸ ἀργύριόν σου σὺν σοὶ εἴη εἰς ἀπώλειαν (8.20), at the same time functioning to hold the whole speech together as a textual unit.

The thematic content of the speech itself also indicates continuity with Paul’s own speech. Verse 20 suggests that Simon’s crime is one involving greed and deceit. The text implies that Simon desires the ability to bestow the Holy Spirit because of its profiteering potential. Whilst Strelan interprets Simon as a sincere insider who mistakenly thinks he can become more fully integrated in the new movement by means of his money (2004: 213), there is better support for Klauck’s interpretation that Simon is slipping back into old ways (2000: 20). Peter’s insight into the state of Simon’s heart not only implies that Simon’s understanding of the Spirit is misplaced, but that his motivation is wrong.

As with Acts 13.4-12, v. 21 shows that Peter, like Paul, has the ability to see into a person’s heart. The repetition of καρδία draws attention to both Peter’s power and Simon’s true nature. That Simon’s heart is ‘not right before God’ and that his intent requires forgiveness suggest a deep-rooted opposition to the apostles, rather than an earnestly misplaced one (contra Strelan).

The element of excommunication that underlies Acts 13.4-12 is explicit in Acts 8.21. Because Simon is a member of the growing community of believers in Samaria, his chastisement by the community’s leaders has to be publicly demonstrated in order to protect the community’s purity and reputation. Verses 18-19 describe Simon’s attempt to purchase the Holy Spirit, a gift often understood by early Christians as a sign of the community’s inheritance (cf. 2 Cor. 1.22). As a result, Peter declares: ‘You have no part or share in this’ (Acts 8.21). Despite having claimed to have joined the Christian community, Simon’s greed shows him to be an impostor. Peter’s curse functions to publicly reverse Simon’s reputation before the eyes of those who had previously listened eagerly to him (8.10). For the audience of Acts, Simon’s ‘true’ status as an impostor should come as no surprise in the wake of Luke’s characterization of Simon as one who practised the arts of the μάγοι, a label that may have invited suspicion in connection to LXX Dan. 2.2.

Like Acts 13.10, the demonic is also present in Peter’s opponent despite the absence of labels like δαιμόνιον, διάβολος, or σατανᾶς. The absence of such terms leads Strelan (2004: 210-11) and Haar (2003: 209) to reject the reading typified by Johnson and Garrett, namely, that Simon represents satanic opposition (Strelan 2004: 213). For Strelan, the text addresses questions regarding who has authority to perform acts of power, rather than to assert Christian victory over satanic powers. However, a weakness in Strelan’s analysis arises from his neglect of the text’s implicit suggestion that Simon is somehow hostage to malevolent power: εἰς γὰρ χολὴν πικρίας καὶ σύνδεσμον ἀδικίας ὁρῶ σε ὄντα (8.23). This image of passivity to evil is conceptually reminiscent of Jesus’ mission statement (Lk. 4.18) and visually reminiscent of demoniacs such as the Gerasene who is bound with chains (8.26-29).^40^ Like Bar-Jesus, Simon is perceived to be opposed to righteousness (8.23: ἀδικίας; 13.10: ἐχθρὲ πάσης δικαιοσύνης). Whilst the standard labels for demonic powers are not used in the story of Simon magus, lexical and thematic links with Acts 13.4-12 and other exorcism narratives in Luke–Acts suggest that readers should understand Simon, like Bar-Jesus, to be under the influence of demonic forces and in opposition to God; this in turn invites the reader to recognize a theological battle between God and evil underlying the narrative.

### Comparative analysis

Peter’s speech shares a number of motifs with Greek and Coptic curses and also with some crucial elements of Faraone’s second category of binding curses: ‘the prayer formula’ (1991: 10). Although it does not match the category’s most defining characteristic – it is not addressed to God – it does manifest a number of its other aspects. It employs the third person optative, ‘may your silver perish with you’. Its disclosure that it is God who is the true victim of Simon’s crime and its desire for confession also evoke Versnel’s related category of ‘prayers for justice’ or ‘judicial prayers’ (1991a: 60-61; 1991b: 192).

In ‘prayers for justice’ the speaker typically seeks vindication for some harm, real or perceived, often relating to stolen goods and/or to potential gossip or slander. In these prayers, the petitioner appears to ‘transfer’ the crime onto the deity, employing quasi-legal vocabulary (Versnel 1991a: 71; Gager 1992: 175). The effect of such a move, Gager argues, is to make the recovery of justice ‘a matter of divine rather than merely human concern’ (1992: 175-76). A bronze tablet from Asia Minor, dated 100 bce–200 ce, illustrates how these prayers present the deity as the one dishonoured.

I consecrate to the mother of the gods the gold pieces that I have lost, all of them, so that the goddess will track them down and bring everything to light and will punish the guilty in accordance with her power and in this way will not be made a laughing stock.^41^

The act of ‘handing over’ the culprit to the deity or to some lesser power is the natural extension of the transferral of victimization onto the deity. In the eyes of the spell-caster, divine punishment functions as either irrevocable punishment or as a ‘conditional and temporary means of pressure’ which might bring the culprit to confession or the perceived damage to restitution (Versnel 1991a: 80).

This ‘transfer’ terminology is clearly seen in other words attributed to the apostles; Paul ‘hands over’ (παραδίδωμι) troublesome figures to Satan for limited periods (1 Cor 5.4-5; 1 Tim 1.20). In the case of Peter’s speech, the transfer is implicit, as the apostle simply states that Simon’s crime is against God and the Holy Spirit, using quasi-legal terminology such as ἀδικία. Simon’s response (8.24) acknowledges that he has been moved into the sphere of divine influence.

Peter’s pronouncement also parallels certain ‘prayers for justice’ that desire a confession from the accused (8.22).^42^ In many curse texts, a public confession of guilt, and the humiliation it would bring, is as desirable as the return of stolen possessions because of the honour it would restore to the deity and to the wronged person (Versnel 1999: 153).

In addition to these aspects of ‘prayers for justice’, Peter’s pronouncement shares other motifs with the curse texts. A number of scholars have recognized the lexical overlap of Peter’s curse (8.20: τὸ ἀργύριόν σου σὺν σοὶ εἴη εἰς ἀπώλειαν) with *PGM* IV.1227-64.^43^

ἔξελθε, δαῖμον,|| ἐπεί σε δεσμεύω δεσμοῖς ἀδαμαντίνοις | ἀλύτοις, καὶ παραδίδωμί σε εἰς τὸ μέ|λαν χάος ἐν ταῖς ἀπωλείαις.

Come out, daimon, since I bind you with unbreakable adamantine fetters, and I deliver you into the black chaos in perdition (ll. 1245-48, trans. Meyer).

It should be acknowledged that *PGM* IV.1227-64 is an exorcism spell rather than a curse text. However, this should not deter one from drawing a comparison with Peter’s curse since the exorcism simply involves the client aiming the curse towards the demon rather than towards an individual. Both texts use ἀπώλεια as an appropriate punishment for their adversary and favour sibilant phonetics in their speech.^44^ The sense of a demonic presence in Peter’s reference to ‘bonds’ also parallels many of the spell texts that describe their opponents as being possessed by a demonic power. The belief that a person could be overpowered by evil is present in both groups of texts.

The style of Peter’s curse on Simon and his money also parallels Greek curses against an individual’s property as well as against their person. The client of *PGM* XL.1-18 curses her daughter’s father who has apparently robbed her daughter of her funeral gifts.

τῆς δὲ | καταβοιῆς ἐνθῦτα κειμένης, κακῶς ἀπολλύοιτο κἐγ γῆι κἐν θαλάσσηι καὐτὸς | καὶ τὰ αὐτοῦ ὑπὸ τοῦ Ὀσερ[ά]πιος καὶ τῶν θεῶν τῶν ἀμπ᾽ Ὀσερᾶπι καθημένων […]

As long as my cry for help is deposited here, he and what belongs to him should be utterly destroyed badly, both on earth and on sea, by Oserapis and the gods who sit together with Oserapis […] (ll. 5-7, trans. Hock).

The speaker of this curse adapts the punishment to the theme of the crime; her adversary is a thief, therefore his possessions are involved in his retribution. A similar style is employed in the first line of Peter’s curse.

Like historiola in the curse spells, Acts 8.4-25 consciously associates itself with historic instances of divine power. Both Peter’s instruction and Simon’s subsequent petition are pregnant with allusions to the OT. Peter’s instruction to repent and Simon’s positive reaction parallel the interaction between Jonah and the city of Nineveh (LXX Jon. 3.1-9), another narrative in which a man of God is sent to a Gentile land to proclaim the news of God’s judgment. Connections include ἀπώλεια (Acts 8.20; Jon. 3.9), μετανοέω (Acts 8.22; Jon. 3.9-10) and κακία (Acts 8.22; Jon. 3.10). The second part of Simon’s petition is also evocative of the words of the narrator in Jon. 3.10. For an audience familiar with the Septuagint, these parallels frame Peter as a prophet in the mould of Jonah, who is sent by God to a non-Jewish audience. These OT allusions function to associate the apostles with the authority of the Jewish scriptures, an important strategy for presenting the apostles’ curses as legitimate acts of power. The text presents Peter in line with the prophets, authoritative agents commissioned to communicate God’s will to his people. Peter’s curses are therefore legitimate in the same way that the prophets were authorized to curse on behalf of the Lord (Jer. 19.1-11; Amos 1.2–2.16). Such strategies for presenting the apostolic curses as carrying divine authorization are vital for defending the apostles’ actions against charges of magic.

An obvious problem with interpreting Peter’s words to Simon as a curse is that its effects do not appear to materialize. Strelan, one of the few scholars to interpret Peter’s words as a curse, argues that Simon’s request for a prayer represents a request for a remedial blessing in a fashion reminiscent of Judg. 17.1-4 (2004: 215). Strelan’s reading is convincing, strengthened by the thematic and lexical parallels between these two narratives.^45^ However, by comparing Peter’s words with Greek curse texts from the ‘magical’ papyri, another dimension to Simon’s reaction can be offered. Peter’s use of χολή connects his speech to a number of texts in the *PGM* where χολή is frequently employed in lists of ‘magical’ ingredients to denote the gall or bile of animals required for the performances of spells.^46^ *PGM* XII.401-44 even acknowledges that such ingredients were inscribed on religious statutes by temple scribes so that common people would not use them to practise magic. The use of χολή, when combined with δεσμός, a term that also has ‘magical’ connotations (binding spells were known as κατάδεσμοι), suggests that Peter’s curse could involve a knowing slur on Simon’s association with magic (*LSJ*: 380).

However, Peter’s use of χολή also brings it into comparison with *PGM* IX.1-14, a text focused on defeating an opponent’s anger.

θυμοῦ σε πα<ύ>σω καί σε πραΰνω χολῆς. ἐλθὲ καὶ διακράτει σιγῇ σιγὴν ϕέρων| τε πα<ῦ>σιν καὶ θυμοὺ<ς> στῆσον ψυχῶν πάντων ὀργάς τε πάσας σβέσον, ϕρένας ὁρκρίσας […]

I’ll give you rest from wrathAnd soothe your raging.Come silently and bringSilence and keep it.Stop ev’ry wrath in soulsAnd melt all angerOf those with temper […] (ll. 12-13, trans. O’Neil).

Spells against anger in the ‘magical’ papyri combine positive sentiments that express the desire to cure an opponent with negative sentiments aimed towards their anger.

κατυπόταξον, καταδοῦλωσον, ϕίμωσον τὴν ψυχήν, τὸν θυμὸν <τοῦ δεῖνα>

Bring into subjection, enslave, and put to silence the soul, the wrath [of him, NN] […] (*PGM* IX.9, trans. Hock).^47^

Other spells on anger use the term θυμοκάτοχος and its cognates to speak of ‘restraining anger’. Like *PGM* IX.1-14, Peter’s words to Simon combine typical curse language with more positive sentiments to see his opponent relieved of his χολή. If we view Peter’s words as a curse on his opponent’s anger, then Simon’s repentant response is in fact the *fulfilment* of Peter’s curse. Interpreting Peter’s words as something similar to a curse against anger would solve the scholarly debate over why the narrative ends before the consequences of the curse are manifested (Garrett 1989: 73). That Peter seems to engage with traditions seeking to affect an opponent’s behaviour fits with a particular theological perspective in Acts which emphasizes repentance and hope for the Gentiles – an attitude at odds with some curse texts which beg the deity not to listen to an adversary’s potential repentance.^48^ In parallel with the response of Nineveh in the story of Jonah, Simon’s repentance is sufficient to defend God’s honour.

## Ananias and Sapphira (Acts 4.32–5.11)

Finally, we arrive at one of the most debated episodes in Acts.

**Table:** 

5.3a	εἶπεν δὲ ὁ Πέτρος ‘Ανανία,
5.3b	διὰ τί ἐπλήρωσεν ὁ σατανᾶς τὴν καρδίαν σου,
	ψεύσασθαί σε τὸ πνεῦμα τὸ ἅγιον καὶ νοσϕίσασθαι ἀπὸ τῆς τιμῆς τοῦ χωρίου;
	(Accusation of demonic association and incrimination of actions)
5.4a	οὐχὶ μένον σοὶ ἔμενεν καὶ πραθὲν ἐν τῇ σῇ ἐξουσίᾳ ὑπῆρχεν;
	(Declaration of responsibility)
5.4b	τί ὅτι ἔθου ἐν τῇ καρδίᾳ σου τὸ πρᾶγμα τοῦτο;
	(Accusation against motives)
5.4c	οὐκ ἐψεύσω ἀνθρώποις ἀλλὰ τῷ θεῷ.
	(Accusation against behaviour and implicit verdict)

Again, Peter’s speech against his adversary is direct. The second person pronoun and the possessive adjective are employed five times in the forms of σου, σε, σοι, σου and σῇ, focusing attention firmly on his opponent. He addresses Ananias by name, which functions to isolate Peter and Ananias from amongst the onlookers. That it is Peter who actively names Ananias (rather than the more balanced option of having Peter speak *to* Ananias) establishes Peter in a dominant position over his adversary.

The structure of Peter’s speech further lends itself to the drama of his judgment. Having named his addressee, Peter, like Paul, employs the use of epiplexis to intimidate his opponent. He asks three questions of diminishing length, heightening the suspense before delivering a simple and syntactically balanced verdict: οὐκ ἐψεύσω ἀνθρώποις ἀλλὰ τῷ θεῷ (5.4). The three rhetorical questions function to communicate Peter’s judgment on Ananias; he is relabelled as demonic, his deviancy is identified, and his status as an impostor is revealed.

The final, declarative sentence does not issue a punitive command, but it is an incrimination that serves to pass Ananias over for divine judgment. The assonance of repeated ω sounds in the words οὐκ ἐψεύσω ἀνθρώποις ἀλλὰ τῷ θεῷ gives the statement its musical quality. Through the structure and style of the text, Acts establishes the potency of Peter’s words; his narration in v. 5 demonstrates its effects.

Like his speech to Simon, Peter’s speech to Ananias employs consonance and sibilance to enhance its rhetorical force. Repeated sigmas across σου, ψεύσασθαί σε and νοσϕίσασθαι ἀπὸ τῆς τιμῆς (5.3), combined with the alliteration of τῆς τιμῆς τοῦ χωρίου, serve to hold the speech together as a distinct unit of text: the repeated phonetics increase its fluidity as it crescendos towards its climax.

The three themes of the two other episodes examined in this article are also present in Peter’s rhetorical questions. First, Ananias is filled by the satan (5.3: reminiscent of Judas in Lk. 22.3), which parallels Peter who is ‘filled with the Holy Spirit’ (4.8). The presence of the demonic also places Peter’s speech in line with the Coptic and Greek curse spells that identify their enemies with demonic activity and wish demonic punishment upon them.

Secondly, impure motives are also present. καρδία is repeated three times in the pericope. Just as Bar-Jesus was said to be ‘full of all deceit and villainy’ (13.10), so Ananias’s heart is ‘*filled* … to lie’ by the satan. The third aspect shared with the other speeches is its excommunicatory function.^49^ Ananias is described as laying ‘only a part’ (μέρος) of his proceeds at the apostle’s feet (5.2). Ironically, the text implies that Ananias dies at Peter’s feet and makes it explicit that Sapphira ends up there too (5.10), the same place where the money was laid. In this way, the punishment – as in some curse texts – mirrors the crime. In bringing only a part of the proceeds to the apostles, Ananias and Sapphira lose their part in the community of believers.

Whilst Peter’s speech itself does not include any explicit demand for divine striking, the effect of his words is one that suggests that his adversaries have been struck by the invisible hand of God.^50^ The text emphasizes this effect through the repetition of πίπτω in the description of Ananias and Sapphira immediately falling down (5.5, 10).

The curse-like nature of Peter’s words is more explicit in his speech to Sapphira. With regard to the narrative of the episode, Peter’s utterance comes at a point when he has seen the effects of his words on Ananias and has waited three hours for Sapphira’s return (5.7). Peter incriminates her for the same crime as Ananias, but his verdict carries an even stronger tone of judgment.

**Table:** 

5.9a	ὁ δὲ Πέτρος πρὸς αὐτήν,
5.9b	τί ὅτι συνεϕωνήθη ὑμῖν πειράσαι τὸ πνεῦμα κυρίου;
	(Incrimination of actions)
5.9c	ἰδοὺ οἱ πόδες τῶν θαψάντων τὸν ἄνδρα σου ἐπὶ τῇ θύρᾳ καὶ ἐξοίσουσίν σε.
	(Verdict)

Peter again expresses his incrimination of Sapphira as a rhetorical question (5.9b). He identifies God as the victim of his opponent’s deceit. Beginning with an emphatic ἰδοὺ̀, Peter’s verdict employs notable sibilance in the words καὶ ἐξοίσουσίν σε to end his curse with a sharp, staccato flourish. The speech is then immediately followed by its fulfilment, as Luke portrays the effect by employing the modifier ἔπεσεν δὲ παραχρῆμα (5.10). Such emphasis on immediacy characterizes Peter’s speech as words of power that cause Sapphira’s death.

If the audience understands Peter’s words to Sapphira as a curse, then it follows that Peter’s words to Ananias should be understood in a similar light. The first incident mirrors the second; both husband and wife are accused of the same crime, both suffer the same punishment, and both are carried out by the young men. The effects of Peter’s words are immediate in both encounters: in v. 5 the text’s use of participles (‘hearing’ and ‘falling’) draws the audience’s attention to the aspects of cause and effect.

### Comparative analysis

Peter’s ‘verdict’ on Sapphira is even more intriguing when it is compared with ‘persuasive analogy’, the third style of curse in Faraone’s typology. These curses reflect a belief that a ritual action might ‘urge’ the victim to become like something to which it is dissimilar (Faraone 1991: 8). Attributes and analogies typically include the coldness of the lead tablets on which *defixiones* are written, or the senselessness of an accompanying figurine.^51^ Often, the characteristics of the corpse by which a curse was buried were also invoked: ‘Just as this corpse lies useless, so too may everything be useless for NN’.^52^ Jordan explains: ‘That the living should be affected by the conditions of the dead with whom curse tablets are deposited is a common idea in Greek magic’ (1999: 118). In Acts 5.9c Peter declares, in dramatic fashion, that the death of Ananias will be the model for Sapphira’s own fate. Grimly, she falls down just as he fell down, becoming as useless as he is. This parallelism could simply be attributed to Luke’s use of irony or accomplished literary style (Witherington 2001: 214, 219) but it also resembles a popular style of cursing that appears to resonate with the spectators who react with ‘great fear’ (5.5, 11) rather than with conversion.^53^ Intriguingly, Faraone stresses that it is rare for the early Greek *defixiones* to actually intend death on their victim. Instead, they desire the lack of success or immobility characteristic of the corpse (1991: 8). In this context, Peter’s proclamation would represent a curse at the harsher end of the spectrum.

The image of Ananias and Sapphira falling at the feet of Peter (5.5, 10), juxtaposed with the upwards movement of the young members of the community (5.6), also evokes motifs from the Greek curse texts.

καὶ ϕίμωσον, | ὑπόταξον, καταδούλωσον τὸν δεῖνα τῷ δεῖνα καὶ ποίη|σον αὐτόν, ὑπὸ τοὺς πόδας μοι ἔλθῃ. |

Put to silence, subordinate, enslave him, NN, to him, NN, and cause him to come under my feet (*PGM* VII.966-68, trans. Hock).

Both texts use πούς in a similar fashion to express the position of power desired or received by the speaker of the curse. It is an image of submission that illustrates the desired restoration of status.

Finally, like historiola, Acts 4.32–5.11 evokes stories of divine judgment in the Jewish scriptures as a means of claiming authority. Commentators have long noted the parallels between the narrative in Acts and the story of Achan in the book of Joshua (Josh. 7).^54^ Although the appeals in Acts to historical executions of God’s power are not explicit like the historiola of the curse texts, they do connect the narrative to OT stories through underlying allusions, thereby claiming divine authority for the apostles’ actions.

## Conclusion

The common themes that can be identified in the apostles’ speeches strongly suggest that the confrontations with Bar-Jesus, Simon magus and Ananias and Sapphira should be read in continuity with each other. As a group of texts, they reveal important information concerning the attitude of the early church towards deviant and threatening behaviour. In each of these three narratives, the opponents’ crimes are associated with deceit and greed, a tendency that appears to complement Luke’s concern for the poor and destitute. Taken together, the texts also shed light on the nature of accusations of magic: Simon and Bar-Jesus’ involvement with magic is associated with deceit and greed, but it also functions as an ethno-classification that emphasizes the foreign territory in which the apostles find themselves. Literary analysis of the three narratives suggests that Luke crafts each text in such a way that audiences would have heard the apostles’ speeches as dramatic words of power. In contrast to interpreters who downplay the apostles’ responsibility in the drama, this article has argued that the text emphasizes the relation between the apostles’ pronouncements and the subsequent afflictions.

Recent scholarship on the diversity of styles and strategies found in ancient Mediterranean curses has allowed us to see a greater degree of similarity between the apostles’ speeches and ‘magical’ curse spells than has previously been appreciated. Although the typologies of Faraone and Versnel cannot be flatly imposed upon the three texts in question in Acts, they have provided a useful heuristic for identifying aspects of correlation and difference between the two groups of texts. The apostles’ words do not include any obvious binding formulae or *voces magicae*,^55^ whereas they do employ common styles of performance, showing elements of ‘direct curses’, ‘prayers for justice’ and ‘persuasive analogies’. Luke also frames the curses in agonistic contexts – the usual context for binding curses – as Paul and Peter compete with other wonder-workers and benefactors. Their accusations against impure motives mirror the concern for ‘evil thoughts’ and *Schadenfreude* in the curse texts (Versnel 1999: 134-35). The motifs common to both groups of texts, such as striking, blindness and demonic-involvement, suggest that their acts of power are difficult to distinguish from each other on a substantial basis (cf. Reimer 2002). Their shared emphasis on urgency suggests that people in Antiquity regarded words of power as successful based on their immediate effect as well as on the authority of the curser.

Against the backdrop of Coptic and Greek curse spells, the apostles’ speeches in Acts appear more like examples of magico-religious cursing than has previously been acknowledged. Descriptions of ‘prophetic foretelling’ do not do justice to the authority of the apostles to deliver words of power that both bless and curse. In each episode, the *apostle’s claim* that it is God’s reputation that is being challenged places the apostle’s speech in a cultural milieu in which curses were legitimized by the transferral of injury and responsibility for vindication onto the deity. That Peter, and to a lesser extent Paul, allows for his wonder-working rival to repent does not place his speech outside the category of cursing, but identifies it primarily with defensive curses aimed at preventing harm or with prayers that request temporary intervention for the purpose of resolving a crisis. Of all the pronouncements, it is Peter’s speech against Sapphira that appears the most aggressive and potentially deviant because of its premeditation, its fatal verdict, and its use of a verbal technique which resembles persuasive analogy.

Similarities between the ‘miraculous’ and the ‘magical’ further explain Luke’s motivation and strategy for presenting the apostles’ speeches as legitimate acts of power. Luke connects the events to an established religious framework (narratives from Israel’s scriptures), portraying Paul and Peter as genuine men of God who are comparable to the prophets of the Jewish scriptures. Their Spirit-powered signs are superior to anything practised by magicians, and they have the authority to judge those inside the Christian community. Rather than explicitly petitioning the deity for action in the manner of the supplicants of the curse spells, the apostles speak for and about the deity in their capacity as his agents. However, for the reasons stated above, Peter’s curse on Sapphira strains these strategies to their limits, which is reflected in the ‘great fear’ of those who hear about the encounter (5.11). In this episode, Luke must rely on the sheer authority of the charismatic leader within the Christian community (4.30-31) to legitimate his actions.

Smith’s topography of religions (2003) offers a useful framework for situating the results of this study: both early Christian communities and practitioners of magic belong to religions of ‘anywhere’ – social formations distinct from the localized religions of ‘here’ (the domestic sphere) and not formally recognized by the religions of ‘there’ (public institutions). The ambiguity surrounding the legitimacy of the apostles’ curses derives from the audience’s relation to this ‘interstitial space’ (2003: 30). To those inside or attracted to the ‘anywhere’ Christian community, the apostles’ speeches served as legitimate acts of power that validated their gospel over competing claims to authority. To those connected to religions of ‘here’ and ‘there’, these speeches may not have sounded so different from ‘magical’ spells.

## Footnotes

1. I gratefully acknowledge the helpful guidance of Drs Peter Oakes and Todd Klutz, University of Manchester, in the development of ideas for this article. I am also thankful to the anonymous reviewer and the editor of *JSNT* for their valuable feedback. This work was supported by the Arts and Humanities Research Council (grant no. AH/L503903/1).

2. Barrett 1994: 617; Conzelmann 1987: 100, who delineates the curse word, its effect and the public reaction; Haenchen 1971: 400; Johnson 1992: 226; Klauck 2000: 53; Parsons (2008: 189) interprets it in line with OT curses against those who practise idolatry, as in Deut. 28.28-29. For scholars who avoid or simply decline to describe Paul’s speech as a curse, see Carter and Earle 1959: 181; Bruce 1995: 249; Dunn 1996: 175; Gaventa 2003: 193; Malina and Pilch 2008: 91; Larkin 2011: 195.

3. For a range of alternative labels for Peter’s speech against Simon magus, see Derrett 1982: 64; Bruce 1995: 171; Dunn 1996: 112; Klauck 2000: 22; Witherington 2001: 287. Aune (1980), Haar (2003), Pervo (2008) and Keener (2013) represent the minority of modern scholars who have actively pursued, or have at least acknowledged, the texts’ similarities with non-biblical curses from Late Antiquity.

4. See Carter and Earle 1959: 116; Barrett 1994: 414; Haar 2003: 185-89. Keener is aware that Paul’s words could be interpreted as a ‘magical curse’ by a reader of Acts, but suggests that the book’s anti-magical polemic requires that they be understood as a ‘prophetic oracle’ (2013: 2022-23). However, he also refers to Peter’s words to Simon – which could be heard as a curse formula – as ‘an appropriate response to a magician’ (2013: 1531).

5. See Carter and Earle 1959: 72; Barrett 1994: 270. Johnson interprets them as prophetic declarations powerful enough to kill (1992: 92); see also Witherington 2001: 218; Parsons 2008: 75; Larkin 2011: 87; Keener 2013: 1193-94.

6. Bruce even writes: ‘It is absurd to try and make him directly responsible … At this stage Peter had not had much experience in pastoral ministry’ (1995: 103, 107). Witherington is the most recent commentator to suggest natural causes of death for the couple (2001: 216, 218). See also Dunn 1996: 64 and Gaventa 2003: 102-103 who highlight the confrontation between cosmic powers; cf. Richter Reimer 1995: 17 who emphasizes the power of sin. Spencer suggests that the couple die of shame (2011: 68).

7. Haar (2003) downplays the connection between Acts 8.4-25 and 13.4-12; he interprets Simon as a practitioner of wisdom traditions rather than as the more commonly proposed charlatan.

8. The thorough challenging, even dismantling, of the old Frazerian categories of religion and magic has been well documented. Close comparative work in recent decades led by Segal (1981) and Smith (2002) has critiqued the substantial distinction between these categories and argued instead for the redirecting of scholarly attention to the contexts within which accusations of magic are made. Whilst I acknowledge the relevance of the recent arguments by classicists and biblical scholars for the continued value of some of the older categories (Betz 1991; Versnel 1991b; Thomassen 1999), this article will adopt an anthropological focus by focusing on the apostles’ confrontations with social deviance.

9. For some examples from the OT, see Deut. 28, 1 Kgs 17.1, Jer. 19.1-11, Amos 1.2-2.16; from the gospels: Mt. 10.11-15, Mk 11.12-25, Lk. 6.24-26, 10.13-15, 11.43-44. In fact, ritual cursing played an important role in all ancient Near Eastern religions (Ritner 1999: 183).

10. Poupon even claims that by the second and third centuries ce, magic has ‘taken over people’s minds, such that there could be no question of a miracle without further investigation into the magical means by which it happened’ (1981: 85, my translation); Reimer suggests that apostles can avoid grounds for suspicion by showing a lack of personal ambition and by attaching their behaviour to an established religious framework (2002: 247-48); Marguerat adds that the apostles must frame their actions, both verbally and ethically, within Christology (2003).

11. I cite the NRSV throughout.

12. LXX Jer. 33.8, 11, 16; 34.9; 35.1; 36.1, 8; LXX Zech. 13.2.

13. Prov. 15.11; 1 Sam. 16.7; Lk. 11.17; Acts 15.8.

14. Aune distinguishes ‘historical evidence’ from literary/folkloristic evidence which reflects the ‘imaginative embellishment’ of actual practices (1980: 1516-17).

15. See the extensive work of Jordan (1979, 1985, 1994, 1999), the analyses of Versnel (1991a, 1991b, 1999, 2002), Faraone (1991) and the collection by Gager (1992).

16. See Gager 1992: 6-9 for a summary of the degrees of change and continuity in *defixiones* from the classical to the Roman period.

17. Ritner 1995: 3352. Brashear also states: ‘Ancient Egyptian magic anticipates much of what can later be found in Graeco-Roman magic’ (1995: 3391).

18. ‘Defixiones are inscribed pieces of lead, usually in the form of thin sheets, intended to bring supernatural power to bear against persons or animals’ (Jordan 1985: 206). They declare to ‘bind’ or ‘nail down’ their victims (Versnel 1991a: 61).

19. Versnel 1991b: 192; Gager 1992: 24-25, 243-44.

20. Cf. footnote 5 above. See also Witherington 2001: 213, 220; Pervo 2008: 52-53; Keener 2013: 1193.

21. The following represents Faraone’s threefold model of styles of binding spells from Ancient Greece (1991: 10) which has subsequently been adopted by others including Gager (1992: 13).

22. This style is also, less frequently, employed with second person subjunctive and future tense expressions (Graf 1997: 124).

23. Ogden 1999: 5. One deposit was found in the vicinity of the temple of Sulis Minerva at Bath (dated to the third or fourth centuries ce; see *Tab. Sulis*), and a large number in the sanctuary of Demeter at Cnidus (dated to the first or second century bce).

24. I acknowledge Bohak’s warning against ‘superficial parallels’ in cross-cultural comparisons involving Jewish magic (2008: 430-33). However, as the primary setting of Acts is a cross-cultural one (representatives of the God of Israel on a Gentile mission), and our interest is in the reception of the apostle’s words within the narrative and among Christian communities across the Empire, the method adopted in this article seems a reasonable one as long as I accept major points of divergence as well as correlation.

25. Ironically, the apostle’s curses are not so different in substance from the kind of behaviour that attracts the deviant label itself in other social situations (Lk. 11.14-28; Acts 16.18-21).

26. Versnel summarizes the body of opinion that the prayers for justice found in the sanctuary of Demeter at Cnidus were likely to have been fixed to the walls: ‘One continually detects the conviction (among modern scholars at least) that publication was necessary to give some real effect to the tablets’ (1991a: 80). See also Versnel 1999: 145.

27. Haenchen (1971: 304) and Conzelmann (1987: 66) follow Bauernfeind (1939: 84, 127) in noting the similarities between Peter’s words in Acts 8.4-25 and some of the Greek Magical Papyri, particularly *PGM* IV.1248. Haar suggests a connection between Peter’s words and binding spells but denies the prominence of the theme of magic in Acts (2003: 189). Like myself, Pervo observes the phonoaesthetic qualities of Paul’s curse on Bar-Jesus (2008: 326 n. 71).

28. The curse spells are almost all characterized by their demands on the deity to act quickly. *Ancient Christian Magic* (*ACM*) 89 is representative of many a request to ‘Bring judgment on our behalf, quickly’. See also *ACM* 90, 91, 92, 95, 98, 108. The emphasis in curse texts on instant effect suggests that people in the ancient Mediterranean world identified successful curses by their immediate effects.

29. *PGM* IV.467-68; 831-32, trans. Hock, in Betz 1992. All English translations of texts from *PGM* are from Betz 1992.

30. *ACM* 90, trans. Meyer. See also *ACM* 89, 91.

31. Although some (Faraone 1991: 8-9; Gager 1992: 23) would question how literally the language of these curses was interpreted.

32. Trans. Smith. See also *ACM* 101, 106.

33. One owner of *defixiones* found in Well V of the Agora at Athens appears to favour deities associated with darkness who can inflict darkness on his adversaries. See Jordan 1985 for expressions such as ‘your dark air’, ‘unilluminated air of oblivion’ and ‘blacking out’.

34. A performer of binding curses from Attica in c. early fourth century ce pronounced: ‘Whoever put a binding spell on me … I put a reciprocal binding spell on all my enemies’. See Jordan 1999: 117, §1 (Inv. G.514.3).

35. Bovon neglects this element in his excursus on the Devil in Luke (2002: 141-42) but is correct to observe that repentance (μετανοέω) is the means of escaping demonic influence, as we shall see in Acts 8.24.

36. The Gerasene demoniac expects to be punished by being sent to the ‘abyss’ (Lk. 8.31). We also find this belief in the devil as an instrument of punishment in the Pauline corpus (1 Cor. 5.5, 13; 1 Tim. 1.20).

37. *ACM* 28; see also *ACM* 88.

38. Jervell’s reading that the proconsul is in fact a ‘Gottesfürchtiger’ (1998: 347-49) is somewhat speculative, with the abundance of Septuagintal allusions in Paul’s curse being better explained by Luke’s apologetic strategy to legitimize the apostles’ actions by connecting them to an ‘established religious framework’ (Reimer 2002: 247-48).

39. Haar (2003: 70-71, 193) is right to point out that Luke’s use of μαγεύω alone is not enough to assume that Luke considers Simon a μάγος in a pejorative sense rather than as a respected professional. However, the agonistic context of the encounter combined with the multiple allusions to magical practices in Peter’s accusation of Simon strongly suggest that some pejorative sense is indeed intended in his depiction of the μαγεύων. In addition, Downing (2003) makes a good case for arguing that Luke’s implied audience is a sceptical one who, I would argue, would have likely felt at home amongst the Jewish, pagan and Christian writers who employed μάγος and its related terms as pejorative ‘stamps’ amongst the ‘divers moyens par lesquels *les miracles* du prophète peuvent être discrédités’ (Heintz 1997: 101). For further discussion of Acts 8.9, see Marguerat 2003.

40. ἀπώλεια (Acts 8.20a) further evokes the threat of subterranean punishment (Lk. 8.31) in this exorcism story.

41. Trans. Versnel 1991a: 74. For the *editio princeps*, see Dunant 1978: 241-44. The *PGM* also contains ‘slander spells’ against those who have dishonoured the gods by misusing sacred objects. *PGM* LVIII.1-14 curses a man who has eaten ‘the sacred fish’, and IV.2471-95 refers to one who claims that Selene drank human blood.

42. Versnel 1999: 152-53, citing text 1 (19-28) in Audollent 1904 (*DTAud*).

43. Bauernfeind 1939: 84, 127; Conzelmann 1987: 66; Haenchen 1971: 304.

44. A number of the *defixiones* found in the Athenian Agora also favour the related term ἀπόλλυμι as an expression of divine punishment. See the curses against wrestlers and racers (IL. 955, 957) and personal rivals (IL. 952, 1000) in Jordan 1985.

45. Both texts employ ἀργύριον.

46. *PGM* IV.46; VII.191; XII.424; XXXVI.284.

47. See also *PGM* VII.940-68; X.24; XII.179-181; XXXVI.35-68.

48. For example, see *IDélos* 2531, *DTAud* 1 (19-28), cited by Versnel 1999: 141, 153. Versnel elsewhere suggests that ‘prayers for justice’ seek one of two types of divine intervention: either irreversible punishment of illness or accident, or ‘conditional and temporary means of pressure’ (1991a: 80).

49. In this respect my reading concurs with that of Havelaar (1997), who interprets the narrative as an excommunication ritual presented in the literary style of a punishment rule.

50. Contra Witherington (2001: 219 n. 91) who uses the lack of explicit striking to play down the likelihood of a punitive miracle.

51. *PGM* XXXVI.231-55 is a typical example of this type of curse. κύριοι ἄγγελοι, ὥσπερ ὁ βά|θρακος οὕτος καταρρεῖ | καὶ ξηρένεται, οὕτως | καὶ τὸ σῶμα τοῦ δεῖνα, τὸ || ἔτεκεν ἡ δεῖνα … Supreme angels, just as this frog drips with blood and dries up, so also will the body of him, NN / whom NN bore … (ll. 246-50, trans. Hock).

52. *DTAud* 68 (Attic, fourth century bce, trans. Faraone 1991: 7).

53. Frankfurter’s thesis that it is the ‘terrifying ambivalence’ of charismatic authority to bless or to curse that demonstrates their efficacy (2005: 184-85) adds a further important layer to Spencer’s inquiry into the source of fear in Acts 5 (2011).

54. For a recent example, see Park 2007: 132. See Marguerat 2002: 155-78 for Gen. 1–3 as the primary allusion behind the narrative of Acts 4.32–5.11.

55. See Aune 1980 and Marguerat 2003 for contrasting views on the name of Jesus as a magical name.
